# Oxidised palm oil and sucrose induced hyperglycemia in normal rats: effects of *Sclerocarya birrea* stem barks aqueous extract

**DOI:** 10.1186/s12906-016-1009-0

**Published:** 2016-02-03

**Authors:** Florence Tsofack Ngueguim, Eloi Christian Esse, Paul Désiré Djomeni Dzeufiet, Raceline Kamkumo Gounoue, Danielle Claude Bilanda, Pierre Kamtchouing, Théophile Dimo

**Affiliations:** Laboratory of Animal Physiology, Department of Animal Biology and Physiology, Faculty of Science, University of Yaounde I, P.O. Box 812, Yaounde, Cameroon

## Abstract

**Background:**

Consumption of foods rich in carbohydrates and fats, result in an increase in obesity and consequently type 2 diabetes. The present study was carried out to evaluate the effects of oxidised palm oil and sucrose (SOPO +S) on some metabolic parameters and to investigate the effects of aqueous extract from barks of Sclerocarrya birrea on SOPO + S induced damages.

**Methods:**

During 16 weeks, animals received every day a supplement of oxidised palm oil (10 %) and 10 % sucrose as drinking water). Control rat received standard diet and drinking water without sucrose. At the end of this period, animal presenting intolerance in glucose test and insensitivity to insulin were continuously feed with hypercaloric diet along with the administration of the plant extract (150 or 300 mg/kg) or glibenclamide (10 mg/kg) during three weeks. OGTT was performed; insulin sensitivity was assessed by performing insulin tolerance test and determining insulin sensitivity index (Kitt). Several parameters were evaluated including body weight, abdominal fat mass, blood glucose levels, blood pressure, serum lipid profile, and serum transaminases (ALT and AST). Oxidative parameters were measured by MDA levels, nitrites levels, SOD levels, reduced glutathione content and by enzyme activities of SOD and catalase.

**Results:**

Animal receiving a supplement of oxidised palm oil and sucrose showed hyperglycaemia, glucose intolerance, insulin resistance and a significant increase in body weight and abdominal fat mass compared to normal rats. In addition, there was a significant increase of SOD in aorta and heart, nitrites in liver and kidney, malondialdehyde (MDA) in heart, liver and kidney. It was also observed a significant reduction in the activities of the SOD and catalase in liver, kidney and reduced glutathione levels in heart. Concomitant treatment of plant extract with SOPO + S brought glycaemia and blood pressure towards normal value, restored glucose tolerance and insulin sensitivity. The plant extract prevent the increase or decrease in the activity of the enzyme depending to the organ, reduced MDA and nitrites levels.

**Conclusion:**

These results highlighted the hyperglycaemic and oxidant character of SOPO + S diet and confirm the hypoglycaemic, and antioxidant action of sclerocarya birrea aqueous extract in diabetes.

## Background

The change in lifestyle including physical inactivity and excess calories, especially the consumption of food rich in carbohydrates and fats, result in an epidemic increase in obesity and consequently type 2 diabetes. In fact, obesity and excess free saturated fatty acids are partially responsible for the inflammatory processes with insulin resistance development which is a major risk factor of type 2 diabetes [1] also, an independent risk factor for high blood pressure. In Africa, particularly in Cameroon many people warm oil for frying (fish, chicken, plantain, irish potato etc…). This warmed oil is used many times until it finishes. This procedure provokes the oxidation of monounsaturated and polyunsaturated fatty acids, increasing the rate of fats which contribute to cytotoxic fatty acid accumulation and the alteration of insulin response [2]. Diabetes mellitus refers to a heterogeneous group of metabolic diseases characterized mainly by hyperglycemia resulting from a lack of secretion and/or action of insulin [3]. This affection is always associated with vascular complications including cardiovascular disorders like high blood pressure. Indeed, type 2 diabetes mellitus (T2DM) increases the risk of developing atherosclerosis in particular due to the increase of blood triglyceride, Low density lipoproteins-cholesterol (LDL-c) and low high density lipoproteins- cholesterol (HDL-c) levels [4]. Despite the development of new drugs and their validation by scientific criteria, the research still continues in scientific community around the world to evaluate antidiabetic activity of raw material isolated from natural products without adverse effects. *Sclerocarya birrea* (*S. birrea*) also called Marula or elephant tree is used to manage various diseases including diabetes. Phytochemical studies have revealed the presence of some compound groups such as of alkaloids, anthocyans, flavonoids, tannins and saponosides [5]. Experimental studies have shown that *S. birrea* exerts its hypoglycaemic activity in streptozotocin-induced diabetic rats [6, 7]. In addition, it has been demonstrated that; aqueous extract from stem-barks of *S. birrea* reduces blood glucose in streptozotocin and nicotinamide-induced diabetic rats by the ability of the plant extract to induce insulin secretion [8]. Despite these studies of *S. birrea* plant, using chemical drugs to induce diabetes, we have investigated the effects of *S. birrea* in hypercaloric food consumption. Firstly, we evaluated the effects of chronic use of oxidised palm oil and sucrose on metabolic parameters in normal rats and evaluated the effects of aqueous extract of *S. birrea* on these parameters.

## Methods

### Animals

Two months old male albinos Wistar rats, weighting between 115 g and 125 g were used to start the experiment. Animals were raised in the Animal House of the Faculty of Science, University of Yaounde I. They were maintained in a temperature room (22 ± 2 °C) on a 12 h light-dark natural cycle. Rats were fed with standard diet and water *ad libitum*. These studies were conducted with the approval of the Cameroon National Ethical Committee (Ref n^o^.FW-IRB00001954).

### Plant material

Fresh stem-barks of *Sclerocarya. birrea* (A. Rich.) Hochst. (Anacardiaceae) were collected in Maroua (Far-North region, Cameroon). The plant was authenticated at the National Herbarium, where a voucher specimen N^o^7770 was deposited. The decoction was carried out by boiling 300 g of powder in 3 L of distilled water for 30 min following the instruction of the traditional healer. The mixture was cold then filtered through Whatman n^o^ 3 filter paper. The filtrate was evaporated in an oven (40 °C) yielding 37.8 g (12.6 %) of a dark-brown *S. birrea* extract.

### Induction of hyperglycemia with oxidised palm oil and sucrose

Normoglycaemic rats were divided into two main groups: one group (7 rats) received standard diet and another group (28 rats) received standard diet supplement with 10 % of oxidised palm oil and 10 % of sucrose in distilled water. Different rats received the diet during 16 weeks. During this period body weight, food and water consumption were estimated weekly. Serum glucose levels of each rat was assessed at the beginning of the experiment (W_0_) then after eight weeks (W_8_) and sixteen weeks (W_16_), using the glucose oxidase method (Accuchek glucometer, Boehringer Mannheim, Germany) [7, 9]. At the end of this period, animals with a blood glucose levels ≥ 126 mg/dL were subject to oral glucose tolerant test (OGTT) and insulin sensitivity test (IST) according to the method of Tritos and Mantzoros [10]. Insulin sensitivity index (K_itt_) was also calculated [11].

### Assessment of insulin sensitivity

To evaluate insulin resistance in all animals, insulin tolerance test was performed [10] and insulin sensitivity index was calculated [11]. Briefly, after 12 h of fasting, the glycemia was evaluated (0 h) and animals received 2 UI/kg of insulin and blood was obtained from tail at 10, 20, 30 and 60 min after insulin injection, serum glucose was measured, and the K_itt_ value was obtained using the following formula:

$$ {\mathrm{K}}_{\mathrm{itt}}=0.693\times 100/{\mathrm{T}}_{1/2},{\mathrm{T}}_{1/2}\mathrm{is}\ \mathrm{the}\ \mathrm{half}\ \mathrm{live}\ \mathrm{of}\ \mathrm{plasmatic}\ \mathrm{glucose} $$

### Effects of aqueous extract of *Sclerocarya birrea* on oxidised palm oil and sucrose induced hyperglycemia

Animals showing intolerance in glucose and insensitivity in insulin were divided into four groups made up of five rats each. One group received distilled water (10 mL/kg), another group treated with glibenclamide (10 mg/kg/day) and two groups were given plant extract at the doses of 150 mg/kg/day or 300 mg/kg/day. The plant extract was administered orally and concomitantly with hypercaloric diet (10 % oxidised palm oil and 10 % sucrose solution as drinking water) during three weeks. Glycemia was determined weekly. Body weight, food and water intake were evaluated weekly. After two weeks of treatment, OGTT was assessed, and then two days after, IST was also assessed before continuing the treatment until three weeks.

### Blood pressure measurement

At the end of the experimental period, arterial blood pressure of all animals was measured as described by Bopda et al. [12]. Briefly, experimental animals were anesthetized by intraperitoneal injection of urethane (1.5 g/kg). Arterial blood pressure was measured from the right carotid artery *via* an arterial cannula connected to a pressure transducer coupled with a hemodynamic recorder (Biopac Student Lab., MP35) and computerized.

### Blood analysis

After blood pressure measurement, arterial blood was collected and centrifuged at 3000 g at 4 °C for 10 min to obtain serum (stored at 20 °C until analysis) for biochemical analysis (glycemia, Alanine amino-transferase, Aspartate amino-transferanse, albumin, creatinine triglycerides, total chlolesterol, HDL-c, LDL-c and atherogen index determined by the ratio of total cholesterol/HDL-c) [13]. These parameters were quantified spectrophotometrically according to the commercial instructions for the kits. Abdominal fats were collected and weighed.

### Aorta, heart, Liver and kidney homogenate analysis

Homogenates (20 %) of aorta, heart kidney and liver samples were prepared in Tris–HCl buffer (pH 7.4). Organs were crushed and then the mixture was centrifuged at 3000 g at 4 °C for 20 min. The supernatant was collected and stored at −20 °C until tissue analysis of SOD (Superoxide dismutase), nitrites, malondialdehyde, glutathione and catalase by using standard methods described elsewhere in the literature.

### Statistic analysis

All data are expressed as mean ± standard error mean. Statistical significance was determined by one way analysis of variance followed by the Tukey post-test using software SPSS version16.0. Differences were considered significant at *p* < 0.05.

## Results

### Effects of a supplement of oxidised palm oil and sucrose on body weight

**Fig. 1:**
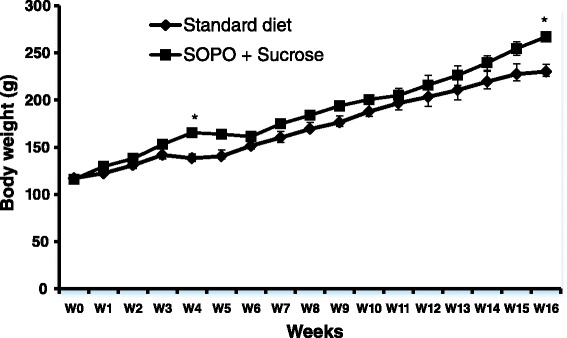
Effects of a supplement of oxidised palm oil (10 %) and sucrose (10 %) on body weight. Each point represents mean ± SEM, *n* = 5. *p < 0.05 vs. standard diet

Figure 1 shows that, food supplement with oxidised oil (SOPO) 10 % and 10 % sucrose (S) in drinking water induced weight gain. At the end of experimental period, animals receiving SOPO diet showed 15.98 % weight gain compared to normal control.

### Effects of a supplement of oxidised palm oil and sucrose on blood glucose

Table 1 depicts blood glucose levels of animals receiving standard diet or SOPO + S. The supplementation with oxidised oil (10 %) and sucrose (10 %) did not increase blood glucose levels after 8 weeks of administration in comparison to standard diet. At the end of the experimental period, animals submitted to SOPO + S presented a significantly increased in blood glucose levels as compared to the animals receiving standard diet and to their respective initial value. The mean glycemia of animals submitted to hypercaloric diet was ≥ 132 mg/dL.

**Table 1:** Effects of a supplement of oxidised palm oil and sucrose on blood glucose

Glycaemia (mg/dL)		
Weeks	Standard diet (*n* = 7)	SOPO + S (*n* = 28)
W_0_	77.60 ± 2.76	78.35 ± 2.76
W_8_	80.60 ± 1.86	84.75 ± 2.64
W_16_	91.40 ± 2.97^a^	133.15 ± 1.97^***C^

Each value represents mean ± SEM. ^a^ *p* < 0.05, ^c^ *p* < 0.001 vs. initial value (at W_0_). ^***^ *p* < 0.001 vs. Standard diet. SOPO + sucrose = standard diet supplement with oxidised palm oil (10 %) and sucrose (10 %). SOPO + S: supplement in oxidised palm oil and sucrose

### Effects of SOPO + S induced-hyperglycemia on Oral glucose tolerance test (OGTT)

**Fig. 2:**
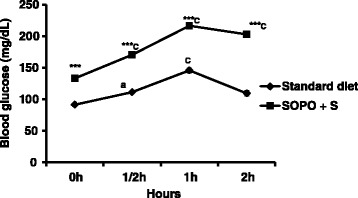
Oral glucose tolerance test in animals receiving hypercaloric diet (SOPO + S). Each point represents mean ± SEM, *n* = 5. ***p < 0.001 vs. standard diet; ^a^ *p* < 0.05; ^c^ *p* < 0.001 vs. initial value

All groups were submitted to OGTT. Figure 2 shows only OGTT in a group of five rats submitted to SOPO + S diet. It was observed that, administration of D-glucose (5 g/kg) to animals induced hyperglycemia to animals receiving standard and SOPO + S diets. Rats submitted to SOPO + S diet, showed a significant increase of glycemia by 48.42 %, one hour after ingestion of glucose as compared to control animals. In addition, the hyperglycemia was maintained during 2 h following the administration of glucose; while it returned to the normal value in control group.

### Effects of SOPO + S induced-hyperglycemia on insulin sensibility

**Fig. 3:**
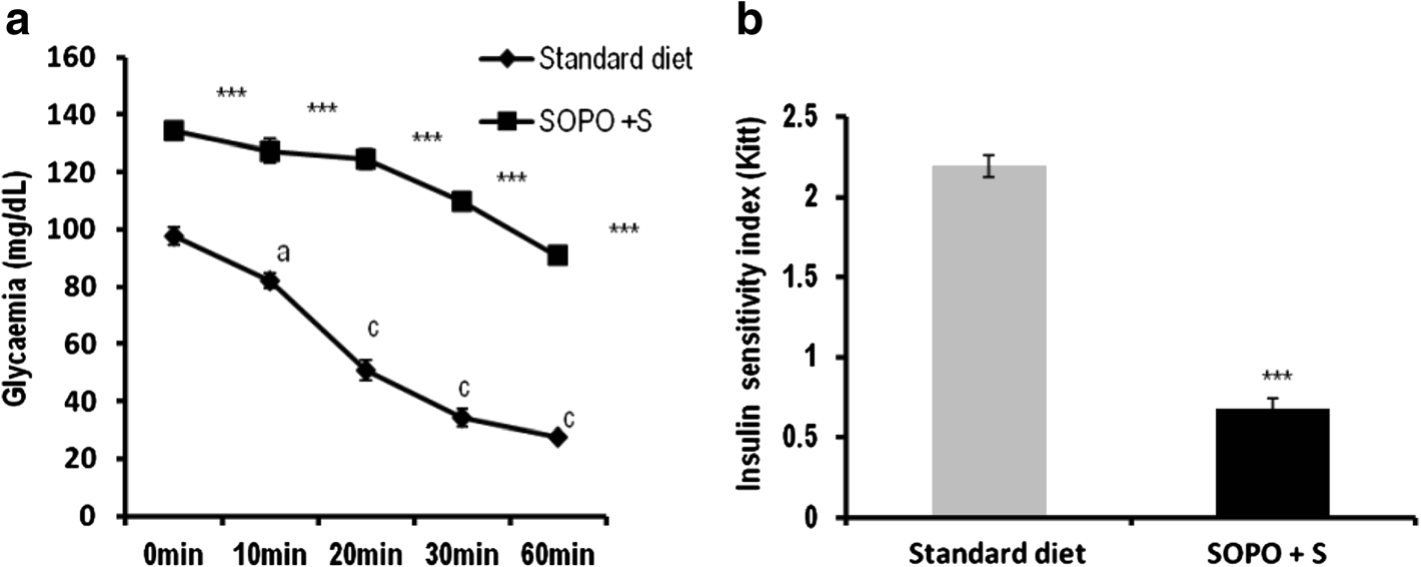
Effect of SOPO+S diet on insulin sensitivity test (**a**) and insulin index (**b**) Each point represents mean ± SEM, *n* = 5. ***p < 0.001 vs. standard diet; ^a^ *p* < 0.05; ^c^ *p* < 0.001 vs. initial value. SOPO + S = supplement in oxidised palm oil and sucrose

Figure 3 represents insulin sensibility test (a) and insulin sensitivity index (b). After subcutaneous injection of insulin (2 UI/kg) to animals receiving SOPO + S diet, the glycemia remained significantly higher throughout the experimental period in comparison with rats receiving standard diet. The insulin sensitivity index was 2.19 for normal rats versus 0.67 for animals receiving SOPO + S diet. Thus, there was a significant decrease of 76.79 % of insulin sensitivity index for animals receiving SOPO + S diet as compared to the control (Standard diet).

### Effects aqueous extract of *sclerocarya birrea* on oxidised palm oil and sucrose induced hyperglycemia

#### Effects on body weight and abdominal fat mass

**Fig. 4:**
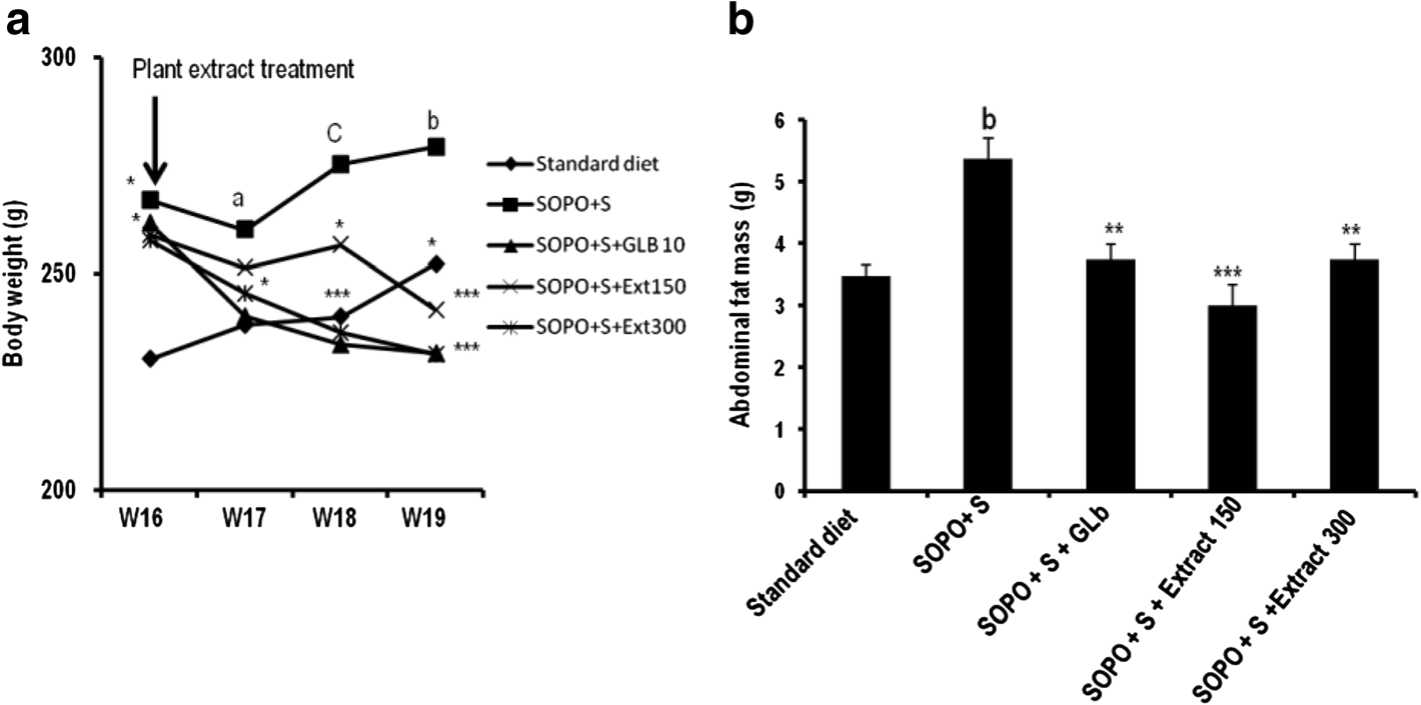
Effects of a concomitant administration of *S. birrea* extract and a supplement of oxidised palm oil and sucrose (SOPO + S) on body weight (**a**) and abdominal fat mass (**b**). Each point or bar represents mean ± SEM, *n* = 5. *p < 0.05 , **p < 0.01 ***p < 0.001 vs. SOPO + S; ^a^ *p* < 0.05; ^c^ *p* < 0.001 vs. Standard diet. SOPO + S = supplement in oxidised palm oil and sucrose

Effects of the aqueous extract of *sclerocarya birrea* on body weight are represented in Fig. 4a. Animals receiving SOPO + S showed a significant rise in body weight from Week17 (W_17_) to week19 (W_19_) in comparison with those receiving standard diet. Concomitant administration of SOPO + S and plant extract during three weeks provoked a significant decrease in body weight of 13.52 % (*p* < 0.01) and 17.17 % (*p* < 0.001) respectively at the doses of 150 mg/kg and 300 mg/kg compared to animals receiving SOPO + S diet. Glibenclamide administered in the same conditions as the plant extract, induced a significant decrease in body weight of 17.10 % (*p* < 0.001) in comparison with the control. Figure 4b showed that, SOPO + S diet increases abdominal fats by 54.07 % (*p* < 0.01) compared to rats receiving standard diet. Concomitant oral administration of the plant extract with SOPO + S diet inhibited the increase of abdominal fats by 44.13 % and 30.18 % respectively at the doses of 150 mg/kg and 300 mg/kg. Glibenclamide administered at the dose of 10 mg/kg also induced an inhibition by 30.21 % of the increase of abdominal fat compared to the control.

#### Effects on food and water intakes

Table 2 shows the evolution of food and water intakes of different groups during the experiment. Supplement of standard diet with oxidised oil and sucrose to normal rats, induced a significant increase in food intake by 13.98 % (*p* < 0.01) in comparison to rats receiving standard diet. When administered plant extract, there was a significant decrease in food intake by 15.95 % and 22.29 % respectively at the doses of 150 mg/kg and 300 mg/kg. Glibenclamide provoked a decreased of 24.55 % of food intake compare to animals receiving SOPO + S diet. At the end of the experiment, there was no significant variation in water intake in different groups.

**Table 2:** Food and water intakes in rats receiving subsequently SOPO + S and aqueous extract of *sclerocarya birrea* during three weeks

Treatment	Food intake (g/rat/day)		Water intake (mL/rat/day)	
	Initial (W16)	Final (W19)	Initial (W16)	Final (W19)
Standard diet	37.08 ± 1.86	43.63 ± 1.42	20.02 ± 0.67	22.95 ± 0.74
SOPO + S	45.72 ± 0.82	49.73 ± 0.65^a^	22.42 ± 0.43	24.43 ± 0.65
SOPO + S + Glibenclamide 10 mg/kg	40.47 ± 0.89	37.51 ± 1.13***	27.48 ± 0.60	23.16 ± 0.70
SOPO + S + Extract 150 mg/kg	40.92 ± 0.77	35.79 ± 0.58***	27.71 ± 0.52	24.16 ± 0.33
SOPO+ Extract 300 mg/kg	42.02 ± 1.08	38.64 ± 0.89***	24.23 ± 0.62	22.67 ± 0.52

Each value represents mean ± SEM, *n* = 5. ^a^ *p* < 0.05, vs. Standard diet, ***p < 0.001 vs. SOPO + S. SOPO + S: supplement in oxidised palm oil and sucrose

### Effects of *S. birrea* on blood glucose levels

Blood glucose levels of different treated and untreated groups are represented in Table 3. Association of oxidised oil and sucrose caused a significant increase in blood glucose levels by 11.21 % (*p* < 0.01) and by 51.96 % (*p* < 0.01) respectively compared to initial value and to normal control (standard diet). Concomitant administration of *sclerocarya birrea* plant extract and supplement of oxidised palm oil and sucrose during three weeks prevented the increase of glycemia. In comparison with their initial value, the decrease in their blood glucose levels was 29.42 % and 35.41 % at the respective doses of 150 mg/kg and 300 mg/kg while it was 35.96 % at the dose of 150 mg/kg and 40.87 % at the dose of 300 mg/kg compared to the rats receiving standard diet. Glibenclamide in the same conditions induced a significant (*p* < 0.001) increase in glycemia by 32.48 % and 38.82 % respectively compared to the initial value and to animals receiving SOPO + S diet.

**Table 3:** Effects of concomitant administration of *sclerocarya birrea* aqueous extract and oxidised palm oil + sucrose on blood glucose in rats

Weeks	Blood glucose (mg/dL)				
	Standard diet	SOPO + S	SOPO + S + Glibenclamide 10 mg/kg	SOPO + S + Extract 150 mg/kg	SOPO+ Extract 300 mg/kg
W_16_	91.40 ± 2.97	132.00 ± 1.71^c^	133.00 ± 1.81^c^	133.20 ± 2.47^c^	134.04 ± 1.69^c^
W_18_	89.20 ± 1.15	149.40 ± 1.03^c$^	102.60 ± 1.99^a$$$***^	104.00 ± 2.30^a$$$***^	107.80 ± 2.15^b$$$***^
W19	96.60 ± 3.24	146.80 ± 1.59^c$^	89.80 ± 2.74^$$$***^	94.00 ± 3.66^$$$***^	86.8 ± 1.85^$$$***^

Each value represents mean ± SEM, *n* = 5. ^a^ *p* < 0.05, ^b^ *p* < 0.01, ^c^ *p* < 0.001 vs. Standard diet. ^$^ *p* < 0.05, ^$$^ *p* < 0.01, ^$$$^ *p* < 0.001 vs. initial value. ^*^ *p* < 0.05, ^**^ *p* < 0.01, ^***^ *p* < 0.001 vs. SOPO + S. SOPO + S: supplement in oxidised palm oil and sucrose

### Effects of *S. birrea* on oral glucose tolerance test, insulin sensitivity and insulin sensitivity index

**Fig. 5:**
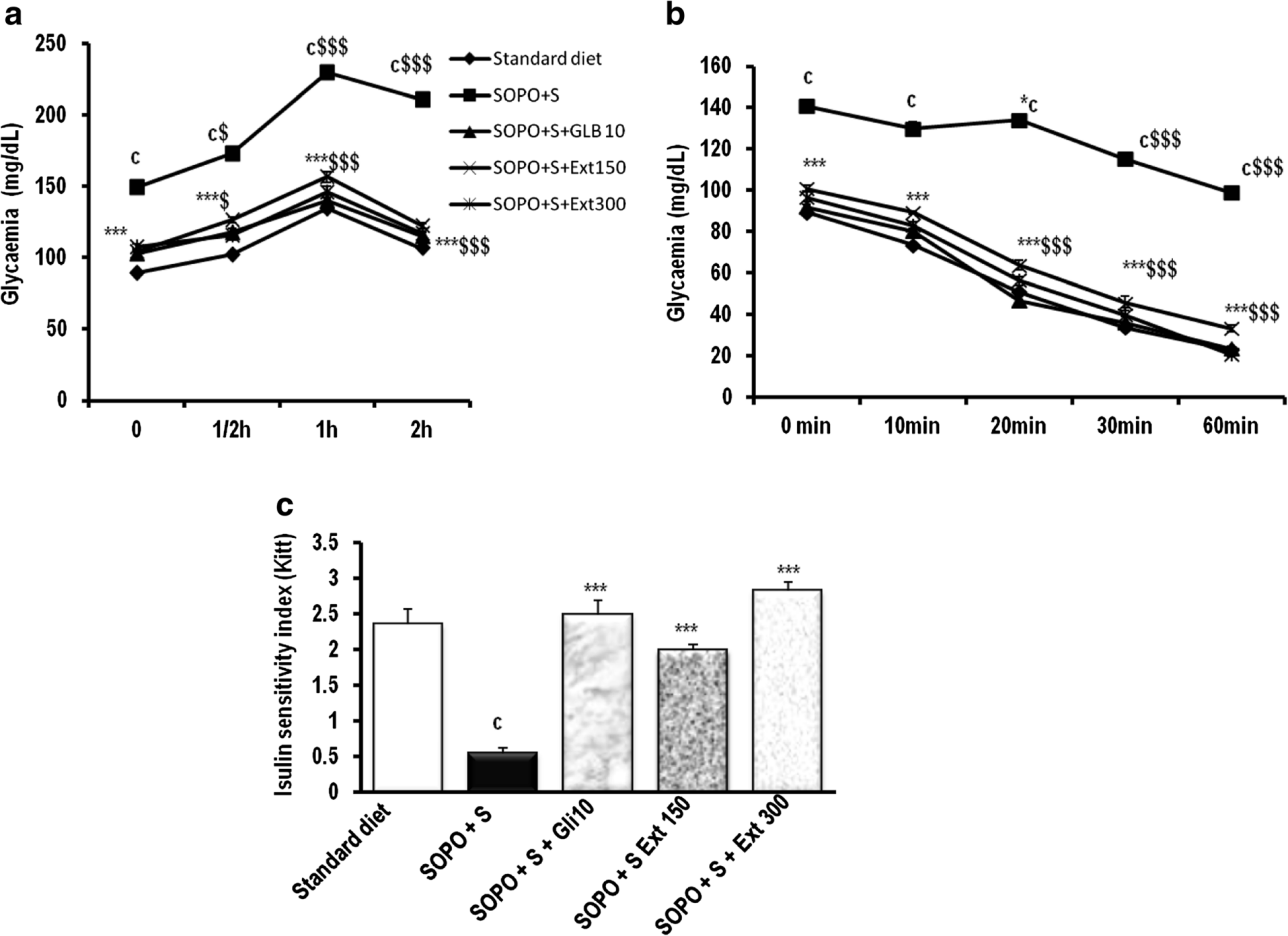
Effects of *sclerocarya birrea* aqueous extract on glucose test tolerance (**a**), insulin sensitivity test (**b**) and insulin sensitivity index (**c**) in rat receiving SOPO + S diet. Each point or bar represents mean ± SEM. *n* = 5 *p < 0.05 , **p < 0.01 ***p < 0.001 vs. SOPO + S; ^a^ *p* < 0.05; ^c^ *p* < 0.001 vs. standard diet and ^$^ *p* < 0.05 ^$$$^ *p* < 0.001 vs. initial value. SOPO + sucrose = supplement in oxidised palm oil and sucrose

Maximal hyperglycemia was observed one hour after the administration of glucose (5 g/kg) in all groups (Fig. 5a). The plant extract administration with SOPO + S diet provoked an inhibition of hyperglycemia induced by glucose one hour after ingestion. The inhibition was 31.73 and 36.35 % (*p* < 0.001) respectively at the doses of 150 mg/kg and 300 mg/kg compared to the control. In the same conditions, glibenclamide induced an inhibition of hyperglycemia by 38.88 % compared to the control. Animals receiving SOPO + S diet showed a high blood glucose level which remains elevated at the end of experiment while animals received concomitantly plant extract and SOPO + S diet showed normal value of blood glucose levels. On the other hand, animals submitted to SOPO + S diet showed no significant variation in insulin sensitivity 20 min following the injection of insulin (Fig. 5b). The glycemia significantly decreased 30 min and 60 min later compared to the initial value. Interestingly, the glycemia remains high throughout the experimental period. Concomitant administration of the plant extract with SOPO + S regimen progressively decrease (*p* < 0.001) blood glucose levels during insulin sensitivity test. The decrease was 37.20 % and 40.20 % respectively at the dose of 150 mg/kg and 300 mg/kg. A supplement of oxidised palm oil and sucrose induced a significant decrease of insulin sensitivity index by 333.33 % (Fig. 5c). The plant extract administered in association with SOPO + S diet induced a significant increase (*p* < 0.001) in the insulin sensitivity index by 264.04 % and by 417.31 % respectively at the doses of 150 mg/kg and 300 mg/kg. Glibenclamide at the dose of 10 mg/kg showed a significant reduction in glycemia by 45 % (*p* < 0.001) 20 min after ingestion of glucose with a reduction of insulin constant value by 355.86 % compared to rats receiving hypercaloric diet.

### Effects of *S. birrea* on blood pressure

Table 4 shows that, association of standard diet with oxidised palm oil and sucrose resulted in a significant increase in systolic blood pressure (SBP) by 63.25 % and the mean blood pressure (MBP) by 63.41 % as compared to normal rats. Simultaneous administration of the plant extract at different doses for three weeks with SOPO + S significantly prevented the increase in systolic blood pressure and mean blood pressure in comparison with the control. The plant extract at the dose of 150 mg/kg induced a significant decrease of SBP and MBP by 44.14 % and 45.12 % respectively. Meanwhile, the plant extract at the dose of 300 mg/kg exhibited a significant decrease of SBP by 44.56 % and by 45.20 % for MBP. In addition the plant extract provoked a significant reduction of the SBP under the normal values. Glibenclamide (10 mg/kg) induced a significant decrease in SBP and MBP respectively by 42.53 % and 41.06 % as compared to the animals receiving standard diet.

**Table 4:** Effects of concomitant administration of *sclerocarya birrea* aqueous extract and oxidised palm oil + sucrose on rat blood pressure

Hemodynamic parameters	Treatment				
	Standard diet	SOPO + S	SOPO + S + GBL 10	SOPO + S+ Ext 150	SOPO + S + Ext 300
DBP (mm Hg)	97.76 ± 2.13	159.50 ± 1.18^$$^	92.60 ± 1.15^**^	86.85 ± 2.5^$**^	87.00 ± 3.40^$**^
MBP (mm Hg)	99.01 ± 2.11	161.4 ± 3.03^$$^	92.90 ± 2.29^**^	88.70 ± 1.84^$**^	88.57 ± 2.88^$$**^
SBP (mm Hg)	101.5 ± 2.09	165.40 ± 2.68^$$^	97.48 ± 1.46^**^	92.39 ± 1.20^$**^	91.70 ± 2.71^$**^

Each value represents mean ± SEM, *n* = 5. ^$^ *p* < 0.05, ^$$^ *p* < 0.01 vs. Standard diet. ^*^ *p* < 0.05, ^**^ *p* < 0.01 vs ^***^ *p* < 0.001 vs. SOPO + S. *DBP* diastolic blood pressure, *MBP* mean blood pressure, *SBP* systolic blood pressure. *SOPO + S* supplement in oxidised palm oil and sucrose. *GBL 10* Glibenclamide 10 mg/kg, *Ext150* Extract 150 mg/kg, *Ext 300* Extract 300 mg/kg

### Effects of *S. birrea* on serum lipid profile and serum glucose

Supplementation of standard diet with oxidised and sucrose contributed to a significant increase of triglycerides (23.84 %), total cholesterol (36.12 %), LDL-c (172.89 %) and atherogenic index ration (106.63 %). The plant extract administered during the last three weeks of experimental period induced a decrease of triglycerides, total cholesterol, LDL-c and atherogenic index as compared to the control. The decrease in triglyceredemia was 37.60 % (*p* < 0.01) and 41.84 % (*p* < 0.001) at the doses of 150 mg/kg and 300 mg/kg. On the other hand, serum total cholesterol decreased by, 15.12 % and 11.26 %, LDL-c by 10.35 % and 36.13 % respectively at the doses of 150 mg/kg and 300 mg/kg. HDL-c which was significantly low (30.11 %; *p* < 0.01) in animals receiving SOPO + S diet, significantly (*p* < 0.001) increased by 27.35 and by 47.00 % respectively at the doses of 150 mg/kg and 300 mg/kg. A significant increase in atherogenic index was observed when compared to normal control. The administration of plant extract at all doses to animals receiving SOPO + S diet significantly reduced these parameters near normal values range (Table 5). A supplement in oxidised oil and sucrose is characterised by a significant increase (50.25 %, *p* < 0.001) in serum glucose compared to normal control. Simultaneous administrations of plant extract resulted in a significant decrease in serum glucose by 21.46 % and by 32.83 respectively at the doses of 150 mg/kg and 300 mg/kg as compared to animals receiving SOPO + S diet. Comparable results were obtained with glibenclamide.

**Table 5:** Effects of concomitant administration of *sclerocarya birrea* aqueous extract and oxidised palm oil + sucrose on lipid profile and blood glucose

Biochemical parameters	Treatment				
	Standard diet	SOPO + S	SOPO + S + GBL 10	SOPO + S+ Ext 150	SOPO + S + Ext 300
Triglycerides (mg/dL)	118.12 ± 2.76	146.29 ± 3.87^$$^	127.51 ± 3.40^*^	124.31 ± 2.45^**^	115.86 ± 4.02^**^
Total Cholesterol (mg/dL)	86.70 ± 3.77	118.02 ± 2.62^$$^	104.37 ± 2.10^$$*^	104.72 ± 3.48^$$^	100.17 ± 3.04^$**^
HDL-cholesterol (mg/dL)	41.06 ± 1.47	28.70 ± 1.81^$$^	49.16 ± 1.73^$$$**^	45.49 ± 2.36^$$^	52.51 ± 1.98^$$**^
LDL-cholesterol (mg/dL)	22.01 ± 5.04	60.06 ± 1.72^$$^	29.71 ± 3.1^**^	34.36 ± 5.24^$$^	24.47 ± 2.45^**^
Atherogenic index	0.24 ± 0.04	0.50 ± 0.01^$$^	0.28 ± 0.02^**^	00.32 ± 0.03^$$^	00.24 ± 0.01^**^
Glucose (mg/dL)	92.99 ± 2.64	139.73 ± 3.2^$$^	90.16 ± 3.32^**^	109.73 ± 2.26^$$**^	93.85 ± 2.06^**^

Each value represents mean ± SEM, *n* = 5. ^$^ *p* < 0.05, ^$$^ *p* < 0.01 vs. Standard diet, ^*^ *p* < 0.05, ^**^ *p* < 0.01 vs SOPO + S

SOPO + S: supplement in oxidised palm oil and sucrose

### Effects of *S. birrea* on hepatic and renal functions

Hypercaloric diet made up of oxidised palm oil and sucrose in association with standard diet induced during 19 weeks a significant increase (*p* < 0.001) in serum transaminase activities (ALT and AST) as shown in Table 6. The plant extract, administered simultaneously with SOPO + S diet, blunted the increase in ALT and AST activities. Serum albumin, significantly reduced in rats receiving hypercaloric regimen, was not significantly increased when administered plant extract at different doses. Glibenclamide provoked a significant reduction of ALT and AST activities, and did not alter serum albumin levels.

**Table 6:** Effects of concomitant administration of *sclerocarya birrea* aqueous extract and oxidised palm oil + sucrose on hepatic and renal parameters

Hepatic and renal parameters	Treatment				
	Standard diet	SOPO + S	SOPO + S + GBL 10	SOPO + S+ Ext 150	SOPO + S + Ext 300
ALAT (UI)	24.68 ± 0.79	43.36 ± 1.24^$$^	26.76 ± 1.87^**^	31.04 ± 2.25^$**^	28.72 ± 1.15^**^
ASAT (UI)	57.16 ± 2.05	78.64 ± 1.50^$$^	67.93 ± 2.91^$*^	66.71 ± 1.62^*^	53.61 ± 2.36^**^
Albumin (mg/dL)	2.27 ± 0.08	1.87 ± 0.07^$^	2.22 ± 0.06	1.98 ± 0.09	1.96 ± 0.07
Creatinine (μg/dL)	2.4 0± 0.10	2.32 ± 0.07	2.43 ± 0.05	2.53 ± 0.03	2.58 ± 0.08

Each value represents mean ± SEM, *n* = 5. ^$^ *p* < 0.05, ^$$^ *p* < 0.01 vs. Standard diet, ^*^ *p* < 0.05, ^**^ *p* < 0.01 vs SOPO + S

SOPO + S: supplement in oxidised palm oil and sucrose

### The effects of *Sclerocarya birrea* on antioxidant parameters

**Fig. 6:**
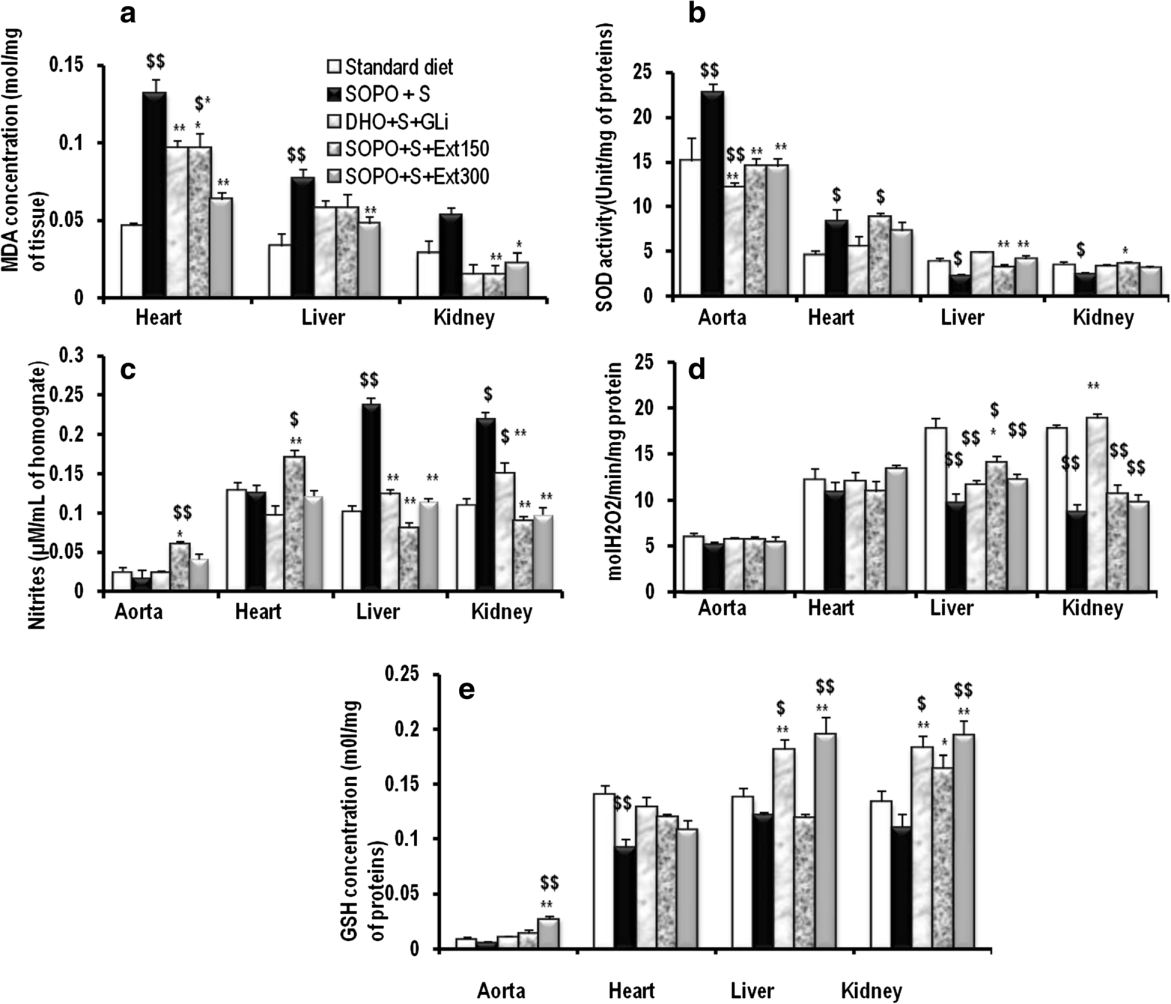
Effects of concomitant administration of *Sclerocarya birrea* and SOPO + sucrose on MDA (**a**), SOD (**b**), Nitrites (**c**), catalase (**d**) and redued glutathione (**e**). Each bar represents mean ± SEM. *n* = 5 *p < 0.05 , **p < 0.01 vs. SOPO + S;. ^$^ *p* < 0.05 ^$$^ *p* < 0.01 vs. standard diet. SOPO + sucrose = supplement in oxidised palm oil and sucrose

At the end of treatment, malondialdehyde, nitrites, reduced glutathione, SOD and catalase activities were analysed. Animals receiving hypercaloric diet during 19 weeks, showed a significant increased in lipid peroxidation by 186 % (*p* < 0.001), 134 % (*p* < 0.001) and by 88.92 % (*p* < 0.01) respectively in the heart, liver and kidney compared to normal control (Fig. 6a). The administration of the plant extract induced a significant decrease in lipid peroxidation by 22.31 % (*p* < 0.001) in heart, 71.46 % in kidney (*p* < 0.01) at the dose of 150 mg/kg and by 49.03 % (*p* < 0.001); 38.69 % (*p* < 0.05) and 58.27 % (*p* < 0.05) respectively in the heart, liver and kidney at the dose of 300 mg/kg. Animals receiving hypercaloric diet are characterized by a significant increase of SOD by 51.28 % (*p* < 0.001) and by 82.55 % (*p* < 0.05) respectively in the aorta and heart in comparison with the control (Fig. 6b). However, a significant decreased (*p* < 0.05) of SOD was noted in the liver (39.60 %) and the kidney (28.41 %). In comparison with animal receiving hypercaloric diet, the administration of plant extract induced a significant (*p* < 0.001) decrease of SOD by 36.02 % and 36.36 % in the aorta respectively at the doses of 150 mg/kg and 300 mg/kg; and a significant increase of 79.89 % of SOD in the liver at the dose of 300 mg/kg and of 44.02 % at the dose of 150 mg/kg in the kidney. A supplement of oxidised palm oil and sucrose was associated with a significant (*p* < 0.001) increase of nitrites in the liver (135.66 %) and kidney (100.64 %), whereas no significant difference was observed in the aorta and kidney (Fig. 6c). The administration of plant extract significantly brought down nitrite levels in the liver by 66.17 % and by 52.22 % respectively at the dose of 150 mg/kg and 300 mg/kg compared to control. In the kidney the decrease was 59.48 % at the dose of 150 mg/kg and 55.94 % at the dose of 300 mg/kg. In the aorta and heart, the plant extract at the dose of 150 mg/kg significantly increased nitrite levels respectively by 265.21 % and 35.39 %. SOPO + S diet also provoked a significant (*p* < 0.001) decrease of catalase in the liver (45.50 %) and heart (51.12 %) compared with normal control (Fig. 6d). Only the plant extract at the dose of 150 mg/kg induced a significant (*p* < 0.05) rise in the catalase levels in comparison with animals receiving SOPO + S regimen. Animals supplemented with SOPO + sucrose showed a significant decrease in reduced glutathione levels, by 34.41 % in the heart (*p* < 0.01) compared to normal control (standard diet) (Fig. 6e). The decrease was not significant in the aorta, liver and kidney. The plant extract administered during the last three weeks induced a significant increase of reduced glutathione. At the dose of 150 mg/kg, the increase rate was 48.18 % in the kidney and 370.94 %, 60.77 % and 76 % respectively in the aorta, liver and kidney at the dose of 300 mg/kg. Glibenclamide (10 mg/kg) induced a significant reduction in reduced glutathione by 39.06 %, 49.03 % and by 65.29 % respectively in the heart liver and kidney.

## Discussion

Excessive intake of fatty acids and or sucrose induced metabolic disorders including lipidemia, insulin resistance and consequently type 2 diabetes [14, 15]. These disorders always cohabit with hypertension. Palm oil is the most important vegetable oils with high oxidant stability fatty acid composition and good plasticity at room temperature [16]. Continual heating of fresh palm oil loses these characters and became harmful to tissues. In fact, palm oil used repetitively provokes the oxidation of monounsaturated and polyunsaturated fatty acids, increasing the rate of fats which contribute to cytotoxic fatty acid accumulation resulting in hypertension and the alteration of insulin response [2]. In addition, diet rich in sucrose resulted in insulin resistance [17]. Combination of oxidised palm oil with sucrose would react synergically to cause abnormalities including glucose intolerance, insulin resistance hyperlipidemia and hypertension and oxidative stress. In the present study, we investigated the effect of a supplement of standard diet with oxidised palm oil (10 %) and sucrose (10 %) in normal rats and the effects of *sclerocarya birrea* aqueous extract on metabolic and cardiovascular damages induced by the diet. Standard diet associated with oxidised palm oil and sucrose during 16 weeks induced body weight gained. The increase in body weight may be due to the presence of fructose, oleic and palmitic acid within the diet. It was also observed an increase in abdominal fats which strengthen the lipogenic character of the diet and explain the hypertrophy and hyperplasia of adipocytes. Animals receiving oxidised palm oil + sucrose were also characterized by hyperglycemia, glucose intolerance and insulin insensibility. According to WHO [18], a fasting blood glucose levels superior or equal to 126 mg/dL, an glucose intolerance with glycemia superior or equal to 140 mg/dL two hours after glucose-load are evocative symptoms of diabetes mellitus. Sucrose can be metabolized into glucose and fructose. The later may be transformed into lipid through lipogenesis “de novo” pathway and in turn induce lipotoxicity [19] and glucotoxicity [4]; preventing insulin action or secretion at cellular levels resulting in a hyperglycemia. In addition, oxidised palm oil increase the level of mono and polyunsaturated fatty acids and thus increase the rate of fats. Thus oxidised palm oil and sucrose contribute to cytotoxic fatty acid accumulation and the alteration of insulin response [2]. In this study, animals fed with a dietary supplement of oxidised palm oil and sucrose presented type 2 diabetes. The insulin resistance observed in this study is related to the increase of abdominal fats [19, 20] and corroborate the insulin sensitivity index (K_itt_) which was dramatically dropped in animal receiving enriched diet with oxidised palm oil and sucrose. Concomitant administrations of the plant extract dose dependently prevented an increase of blood glucose levels in OGTT and IST. These results show that, the plant extract could act at peripheral levels by several mechanisms such as reducing glucose absorption from the gastrointestinal tract and/or stimulating peripheral glucose utilization. Furthermore, during three weeks of administration, the plant extract also exhibited hypoglycemic effect. Our results support those of Makom et al. [8] who demonstrated the hypoglycaemic action of *S. birrea* in a validated animal model of diabetes. They showed that *S. birrea* aqueous extract acts on pancreatic beta cells by enhancing insulin secretion. In addition, previous study revealed that *S. birrea* ethanolic extract increased hepatic glycogen storage [21]. Thus, these different mechanisms could contribute to reduce blood glucose levels observed in this study. It is known that chronic intake of sucrose result in an increase of superoxide anion which suppressed endothelial NO and in turn, damage vascular function. Abnormal glucose metabolism is associated to impaired glucose tolerance, insulin resistance syndrome which is closely related to endothelial dysfunction. This leads to decrease NO availability and enhanced endothelin production, tilting the balance between endothelin and NO production resulting in increased vasoconstriction in thus high blood pressure [22, 23]. In our study, oxidised palm oil and sucrose induced hypertension characterized by an increase in systolic and mean blood pressure. *Sclerocarya birrea* extract administered during three weeks led to the decrease in blood pressure under the normal value. This may be explained by the ability of the extract to reduce insulin resistant syndrome. Hypercholesterolemia and hypertriglyceridemia are involved in the development of atherosclerosis and coronary heart disease which are the secondary complications of diabetes [24]. *S. birrea* significantly reduced serum triglycerides and total cholesterol and LDL cholesterol. We can conclude that, the plant extract could modulate blood lipid abnormalities, suggesting that, the plant extract would be helpful to the prevention of diabetic complications through reduction of dyslipidemia. In our study we observed a significant increase in ALT and AST activities. The increase in these two parameters indicates hepatotoxicity which is related to glucose induce-oxidative stress [25, 26]. Lipids and glucose autoxidation generate radical oxygen species which attack cells and damage their functions (membrane fluidity and membrane bound enzyme) causing the leakage of these enzymes into the blood stream [4, 27]. This hypothesis is strengthened by a significant increase in MDA which is one of lipid peroxidation product. Our study showed that, *Sclerocarya birrea* aqueous extract improved not only serum transminases level but also significantly decreased the concentration of MDA. *S. birrea* could exert its action by inactivating lipid peroxidation reactions and by reducing free radical generation due to its antihyperglycemic action. Permanent hyperglycemia provokes alteration of antioxidant defence system which could increase the deleterious effects of free radicals [28]. In the present study, a chronic administration of oxidised palm oil and sucrose induced a significant decrease of SOD and catalase in the liver and in the kidney. These two enzymes induce respectively decomposition of superoxide anion (O^−^_2_) and hydrogen peroxide. The decrease observed can be due to the inactivation of active site of this enzyme and/or to the increase of the concentration of superoxide anion (O^−^_2_) which can react with NO and form nitrite peroxide (ONOO^−^). This molecule is very toxic and, its formation decrease bioavaibility of NO which in turn increase endothelial dysfunction [29]. Reduced gluthation (GSH) plays an important role by protecting the organism against ROS [30]. The increase in GSH observed can be attributed to the high production of ROS which could lead to the reduction in GSH biosynthesis and/or to its degradation [31]. However oral administration of the plant extract showed a marked increase in the activity of these enzymes suggesting it antioxidant activities in hyperglycemia induced oxidative stress.

## Conclusions

A supplement of oxidised palm oil (10 %) and sucrose (10 %) during nineteen weeks induced glucose intolerance, insulin resistance associated to dyslipidemia, alteration of hepatic function, hypertension and oxidative stress. Concomitant administration of *Sclerocarya birrea aqueous* extract during three weeks dose dependently restored glucose tolerance, lipidemia, and hepatic function; prevent blood pressure and oxidative stress. The present study justifies the traditional used of *S. birrea* for the management of diabetes. Further studies are in progress to elucidate the exact mechanism by which the plant extract exert these activities on diet induced-diabetes.

## Abbreviations

ALT: alanine amino-transferase
AST: aspartate amino-transferase
HDL-c: high density lipoprotein cholesterol
LDL-c: Low density lipoproteins cholesterol
NO: nitric oxide
OGTT: oral glucose tolerance test
IST: insulin sensitivity test
SOPO + S: supplement in oxidised palm oil and sucrose
